# Genetic variability and drug resistance mutations in HIV-1 infected individuals on HAART or drug naïve in Limbe, Cameroon

**DOI:** 10.1186/1742-4690-9-S2-P158

**Published:** 2012-09-13

**Authors:** L Agyingi, D Barengolts, L Mayr, T Kinge, M MBida, P Nyambi

**Affiliations:** 1 New York University School of Medicine, New York, NY, USA; 2 Limbe Regional Hospital, South West Region, Limbe, Cameroon; 3 Faculty of Biomedical Sciences, University of Dschang, Dschang, Cameroon

## Background

Cameroon is a country in West Central Africa with a population of about 20 million inhabitants, with an estimated HIV prevalence of 5.1% in the general population. The HIV epidemic in this region is marked by a broad genetic diversity dominated by Circulating Recombinant Forms (CRFs).

## Methods

To characterize HIV-1 genotypes circulating in HIV positive individuals in Limbe, Cameroon, we phylogenetically analyzed blood samples from 116 HIV positive patients. Of 116 samples tested, 110 were amplified by nested PCR at the Gag, Pol and Env genes. Sequences obtained were phylogenetically analyzed with reference sequences from the Los Alamos database. The RT region of samples was also amplified to identify mutations that conferred resistance to Reverse Transcriptase inhibitors (RTIs) using the Stanford University Drug resistance database.

## Results

Our results revealed a broad genetic diversity, dominated by circulating recombinant forms as follows: CRF02_AG 50.4% (n=54), CRF22_01A1 7.4% (n=8) F2 3.7% (n=4), D 2% (n=2), CRF43_02G 2.8% (n=3), CRF18_CPX 2.8% (n=3) and recombinant forms (RFs) 31.7 %( n=34). Most RFs contained CRF02_AG in one or two HIV loci analyzed. RT sequences of 3 patients on HAART and 15 drug naïve individuals harbored mutations which conferred resistance to RTIs.

## Conclusion

CRF02_AG continues to be the most predominant strain in Cameroon, CRF22_01A1 on the increase. Identification of drug resistance strains in drug naïve patients suggest these viruses are being transmitted in the study population and highlights the need of drug resistance testing before start of ART for HIV patients.

